# State-of-the-Art Advances of Nanomedicine for Diagnosis and Treatment of Bladder Cancer

**DOI:** 10.3390/bios12100796

**Published:** 2022-09-27

**Authors:** Chenfan Kong, Shaohua Zhang, Qifang Lei, Song Wu

**Affiliations:** 1 Department of Urology, The Third Affiliated Hospital of Shenzhen University, Shenzhen University, Shenzhen 518000, China; 2 Graduate School, Shanghai University of Traditional Chinese Medicine, Shanghai 201203, China; 3 Department of Urology, The Affiliated South China Hospital of Shenzhen University, Shenzhen University, Shenzhen 518000, China

**Keywords:** bladder cancer, nanomedicine, diagnostic methods, drugs delivery

## Abstract

Bladder cancer is a common malignant tumor of the urinary system. Cystoscopy, urine cytology, and CT are the routine diagnostic methods. However, there are some problems such as low sensitivity and difficulty in staging, which must be urgently supplemented by novel diagnostic methods. Surgery, intravesical instillation, systemic chemotherapy, and radiotherapy are the main clinical treatments for bladder cancer. It is difficult for conventional treatment to deal with tumor recurrence, progression and drug resistance. In addition, the treatment agents usually have the defects of poor specific distribution ability to target tumor tissues and side effects. The rapid development of nanomedicine has brought hope for the treatment of bladder cancer in reducing side effects, enhancing tumor inhibition effects, and anti-drug resistance. Overall, we review the new progression of nano-platforms in the diagnosis and treatment of bladder cancer.

## 1. Introduction

Bladder cancer is the sixth most common cancer in the United States. Approximately 80,470 new cases of urinary bladder cancer (61,700 men and 18,770 women) were diagnosed in the United States in 2019 with 17,670 deaths (12,870 men and 4,800 women) occurring during this same period [1,2]. In general, bladder cancer can be classified into non-muscle-invasive bladder cancer (NMIBC) and muscle-invasive bladder cancer (MIBC) based on how extensive the tumor is [3]. Approximately 70% of the patients are diagnosed with NMIBC and the rest are MIBC and advanced bladder cancer [4]. Moreover, bladder cancer costs per patient range from 40,000 USD to more than 170,000 USD in the USA, which causes a huge financial burden for patients [5,6].

**Figure 1:**
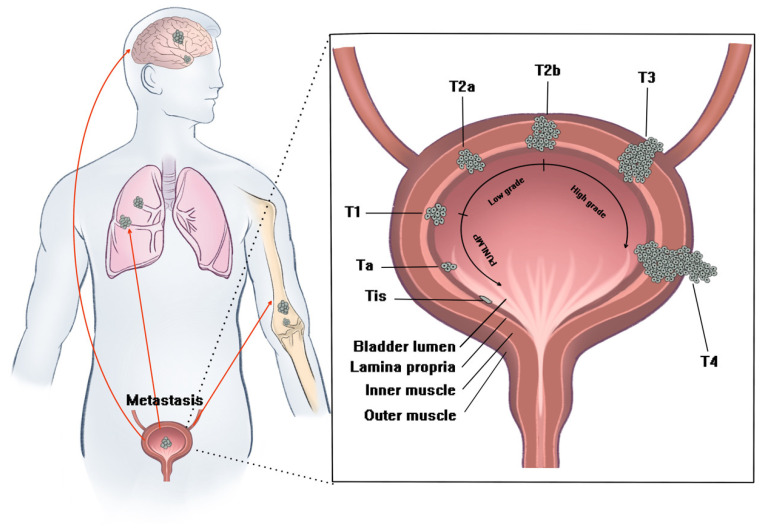
The stage and grade of bladder cancer. Bladder cancer is divided into five stages according to the degree of progression: T0 (Ta: non-invasive papilla-carcinoma and Tis: tumor in situ); T1 (Tumor invades the bladder mucosa); T2 (Tumor invades the bladder inner muscle); T3 (Tumor invades the bladder outer muscle); and T4 (Tumor metastasis). Non-muscle-invasive bladder cancer (NMIBC) refers to T0 and T1 stages, while muscle-invasive bladder cancer (MIBC) refers to ≥T2 stages.

Currently, the mainstream diagnosis methods are cystoscopy, biopsy, and computer tomography (CT) scanning. It is difficult to identify the oblate abnormal tissue and tumor at an early stage by cystoscopy. Therefore, the accuracy of cystoscopy is decided by the examiner’s experience. In this regard, it is useful to evaluate the tumor location and tumor invasion of the surrounding tissue by CT when the tumor is large. Nevertheless, it is difficult to distinguish the T2, T2, and T3 stages precisely [7,8,9]. The treatment and administration of bladder cancer mainly depend on the stage of tumor and the degree of metastasis (Figure 1). For NMIBC, the current standard therapy is complete transurethral resection of the bladder cancer (TURBT) to remove as much of the tumor as possible and then conduct intravesical therapy to prevent recurrence or delay progression to a higher stage [10]. As for MIBC and the advanced stage of bladder tumor, systemic and intraperitoneal chemotherapy, adjuvant radiotherapy, photodynamic therapy as well as targeted drugs are commonly used in clinic to prolong survival time [11]. However, even after taking the full course of standard therapy and enduring all the adverse effects, the recurrence rate of bladder patients is still unsatisfying [12,13,14,15,16]. For example, 40% of NMIBC patients’ stage progressed after BCG instillation [17]. Moreover, the latest study suggested that the effect of chemotherapy for treating advanced bladder cancer was still far from satisfactory. About 44% of patients suffer recurrence or progression despite taking chemo-drugs instillation after TURBT [10,18]. Therefore, there is an unfulfilled need for more precise diagnostic methods and more effective treatments.

At present, there are many difficulties in the application of drugs which are commonly used in the treatment of bladder cancer. For example, many chemotherapy drugs have problems such as large side effects, low bioavailability, poor solubility, and tumor resistance, though with the continuous progress of drug research, some problems have been solved. In clinical practice, both doctors and patients are still expecting better drugs.

Having emerged in the past decades, nanomedicine is the application of nanotechnologies in medicine to improve diagnosis as well as therapy. More specifically, nanobiotechnologies play an important role in the discovery of biomarkers and molecular diagnostics and facilitate the integration of diagnosis and therapy. Typically, nanomaterials have been widely developed for cancer treatment. The flexibility in size, structure, and feasibility of precise release make nanomaterials a promising drug delivery tool. For instance, by packaging with a well-designed nano-shell, the diagnostic agents as well as therapeutic agents can attach to the tumor cell, so as to increase the concentration and prolong the exposure time. Moreover, using a nano-vehicle with a biphasic structure can increase the solubility of hydrophobic agents and improve the drug distribution in the bladder [19,20]. Moreover, solid tumor tissues are rich in blood vessels, wide in vascular wall space, poor in structural integrity, and lack lymphatic return, resulting in high permeability and retention of nanoparticles. That is known as the enhanced permeability and retention (EPR) effect. The EPR effect promotes the selective distribution of macromolecular substances in tumor tissue as well as preventing the internal agents from prematurely exposing to the body fluid or the normal tissue, so as to protect the agents from degradation and decrease the immunogenicity of agents [20]. It is noted that multifunctional nanomaterials have received intense attention recently. These kinds of novel particles can realize the purpose of diagnosis and treatment simultaneously [21,22,23,24]. This review mainly discusses the current nanotechnologies in the diagnosis and treatment of bladder cancer.

## 2. The Current Diagnostic Methods

**Figure 2:**
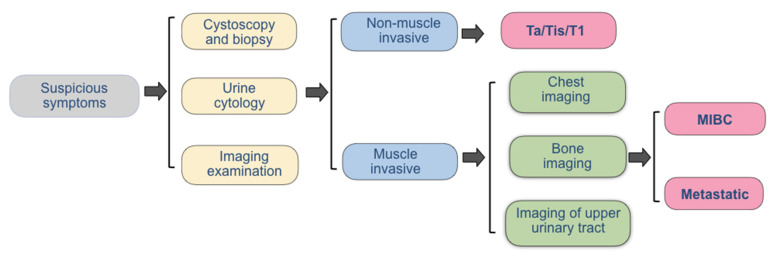
Current strategies for the diagnosis of bladder cancer.

An accurate diagnostic method is critical for a comprehensive assessment of bladder cancer progression and prognosis. Prior to the treatment, the main diagnostic methods of bladder cancer include cystoscopy and imaging examination, which are used for the qualitative diagnosis, location diagnosis, and staging diagnosis of bladder cancer. When a patient has the most typical symptoms of painless hematuria, endoscopic biopsy or biopsy and histopathologic examination are the basis for the diagnosis of bladder cancer (Figure 2). In addition, the clinical stage before treatment can be determined according to the CT examination of the whole body. If liver metastasis, lymph node metastasis, and bone metastasis are suspected, MRI and PET/CT can provide an additional reference in addition to CT results. Additionally, symptom assessment, urine cytology, and biomarkers detection are also commonly used in clinic.

Cystoscopy and biopsy are the gold standards for the diagnosis and recurrence monitoring of bladder cancer. However, frequent cystoscopy is often required, and cystoscopy is an invasive procedure. Therefore, it is possible to cause urinary tract infection, urethral stricture, and other complications [25]. In addition, the detection rate of bladder cancer is closely related to the doctors’ experience. Recently, a fluorescence cystoscope has been applied to diagnose bladder cancer by intravesical instilling 5-aminolevulinic acid (ALA) of hexaminolaevulinic acid (HLA). Compared with a normal cystoscope, a fluorescence cystoscope is more likely to detect tumors, especially carcinoma in situ. However, inflammation, recent TURBT or BCG instillation may cause false positive results. Therefore, there is an urgent need for new technical advances used to assist and improve cystoscopy.

Urine cytology and biomarkers tests are routine methods for the diagnosis of bladder cancer. It is not only non-invasive and has high specificity for the detection of bladder cancer, but sensitivity is related to the type of bladder cancer [26,27,28]. For example, the cytology sensitivity of low-grade bladder cancer is only 16% [29], which limits the clinical application. As for biomarkers tests, numerous proteins and RNA were found related to bladder cancer. However, the FDA only approved six biomarkers for urine tests and no marker test for blood or tissue due to the lack of high-quality prospective trials and low level of clinical evidence [30]. Nevertheless, none of them were sufficiently effective when used in isolation. Further randomized clinical trials are still needed in the future. Therefore, though clinical urine cytology and urine biomarkers may use in individual therapy guidelines, they are still auxiliary means of cystoscopy in clinical practice (Table 1).

Abdominal and pelvic CT is always the first choice of imaging examination for bladder cancer [33]. It can effectively detect bladder cancer and determine its location and stage [7]. For T4 stage bladder cancer, it is possible to accurately determine the location, extent, and depth of invasion of tumor via enhanced CT scan and routine scan combined with multiplanar image reconstruction. However, CT scan cannot illustrate the structure of the bladder wall. Therefore, it is hard to distinguish T1, T2, and T3a stages only using CT scans [9]. MRI is often used to assess the preoperative stage and lymph node metastasis. Generally speaking, using MRI to diagnose a T2 or T3 stage bladder tumor is much better than using CT scan. Especially when diffusion-weighted imaging and dynamic enhancement are used, MRI can better distinguish NMIBC and MIBC [11]. However, to some extent, the imaging quality of bladder wall and tumor depends on whether the bladder is filling. In addition, it is difficult to perform enhanced CT/MRI when patients are allergic to iodine contrast agents or have renal insufficiency [34,35]. Clinically, ultrasound imaging is widely used in the screening of patients with hematuria, especially in patients with iodine contrast medium allergy and renal insufficiency [36]. However, the accuracy and detection rate of ultrasonography are not satisfactory [37].

## 3. The Current Therapies

**Figure 3:**
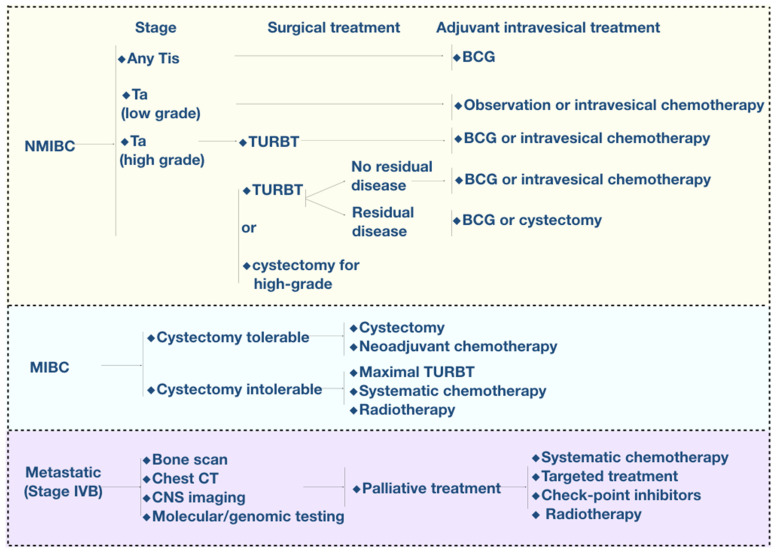
Current therapies of bladder cancer. TURBT: transurethral resection of the bladder cancer; BCG: bacillus Calmette-Guerin; CT: computer tomography.

Current clinic therapies for bladder cancer are illustrated in Figure 3. For NMIBC, TURBT combined with single-dose immediate intravesical chemotherapy (SI) is the current standard therapy [38]. SI with gemcitabine and mitomycin can effectively prevent the implantation of residual tumor cells and reduce the risk of recurrence [13,38]. Furthermore, patients are recommended intravesical chemotherapy and BCG according to their stages. Epirubicin and mitomycin are usually used for intravesical chemotherapy while BCG is another commonly used intravesical agent for NMIBC. BCG was utilized for bladder cancer treatment as early as 1976 [39] and was finally approved by the FDA for NMIBC in 1990. Although decades have passed, intravesical instillation of BCG is still the most mature and widely used immunological therapy for NMIBC. According to the 2020 version of the NCCN (National Comprehensive Cancer Network) clinical practice guidelines on bladder cancer, BCG intravesical instillation is suggested for the induction intravesical instillation of medium-risk patients and should be prioritized for the induction of high-risk patients after TURBT. As for maintenance therapy, BCG is preferred for all medium/high-risk patients rather than chemotherapeutic drugs [33]. After instilling BCG, the fibronectin attachment protein (FAP) expressed on the mycobacterium wall combines with fibronectin expressed on urothelium, which helps BCG attach to the urothelium [40,41,42]. Bladder cancer cells then internalize BCG through micropinocytosis while it is much more difficult for normal urothelium cells to uptake it [43,44,45]. Then, BCG could activate multiple kinds of immune cells and initiate innate immune responses as well as adaptive immune responses [46,47,48]. In addition, BCG could cause bladder cancer cell death directly through apoptosis [49,50,51] and cellular oxidative stress [52,53,54].

As for MIBC and advanced bladder cancer, the standard therapy is neoadjuvant chemotherapy followed by radical cystectomy. Usually, advanced bladder cancer is more sensitive to platinum-based combination chemotherapy. Therefore, the major regimens are methotrexate, vinblastine, doxorubicin and cisplatin (MVAC), and gemcitabine cisplatin/carboplatin (GC regimen). In addition, adjuvant and palliative radiotherapy can be used as treatments for patients who are not suitable for cystectomy to increase the local tumor control rate and prolong the overall survival of the patients [55,56,57]. Immunotherapy and targeted therapy have developed rapidly in recent years. Compared with conventional chemotherapy, PD-1/L1 mAb-based immunotherapy has significantly improved the overall survival rate for advanced bladder cancer [58,59,60]. FGFR inhibitors as well as HER2 inhibitors have been approved for the treatment for advanced bladder cancer [61,62], which brings new hope to patients with chemotherapy failure.

Because the bladder is a hollow organ, the location of bladder tumors, especially NMIBC, is generally more superficial than that of other organs. Therefore, in addition to normal surgical treatment and chemotherapy, it is easier to implement local treatment, such as photosensitizer-based photodynamic therapy (PDT). Researchers have studied photosensitizers since the beginning of last century. These photosensitizers are usually dyers, which are able to accumulate in tumor tissues and are sensitive to light. After the oxygen molecule activation by light, reactive oxygen species (ROS) generate and therefore induce cytotoxic effects. Based on this mechanism, researchers successfully shrunk rats’ malignant tumors by using a photosensitizer with visible light in 1972 [63]. Following up on this study, PDT was widely developed and confirmed to have therapeutic effects on various cancers. The utilization of traditional photosensitizers to eradicate superficial bladder tumors has also been discussed. Regrettably, a limited number of photosensitizers have been approved for clinical use for bladder cancer despite various photosensitizers, mainly including the porphyrins, chlorins, and phthalocyanines as well as their derivatives, having been described in the literature. At present, only photofrin, Levulan, and Deuteporfin have been approved for superficial bladder cancer by the FDA. However, all of the current approved PDT agents have great limitations [64]. The long drug metabolism and increasing skin photosensitivity caused by photosensitizers may cause cutaneous toxicities, thus prolonged light-avoid time is needed. Moreover, the poor tumor selectivity of traditional photosensitizers also limits their use in bladder cancer. Photofrin/profimer sodium-PDT can lesion the adjacent tissue in the muscle layer of the bladder, thereby leading to irreversible bladder fibrosis and contracture [65,66]. In addition, it is necessary to have ample oxygen for ROS generation. Paradoxically, most malignant tumor displays a hypoxic environment within and the PDT therapy itself can cause hypoxia, which eventually reduces the curative effect. Moreover, by activating cellular immunity, PDT therapy can up-regulate the expression of PD-L1 [67,68,69], which leads to immune escape. Due to these drawbacks, PDT has not yet been recommended for bladder cancer treatment in the clinical guidelines.

## 4. Nanotechnology in Diagnosis

### 4.1. Nanotechnology in Light-Based Imaging

It is difficult to detect tiny or flat cancerous tissue accurately with the naked eye, which often results in a high recurrence rate after TURBT. Combining cystoscopes with bladder tumor-specific imaging agents is a good solution for improving diagnostic methods. Semiconductor nanocrystals, also called quantum dots (QD), have gained more attention than before for their unique properties in optics and electronics. Being able to emit fluorescence and carry tumor-targeting molecular agents makes semiconductor nanocrystals promising tumor tracers. It has been reported that, after intravesical instillation of the complex of QD and CD47 antibodies, this nanoparticle significantly concentrated in bladder tumors [70]. The combination of anti-CD47-QD and blue light cystoscopy showed satisfactory diagnostic accuracy for bladder cancer [71]. However, it should be noted that core materials such as cadmium (Cd) and selenium (Se) of QD might have long-term tissue toxicity, though the toxicity of heavy metals has not been observed in vivo. The prostate stem cell antigen (PSCA) is also a biomarker highly expressed on the surface of bladder tumor cells [72]. Yuan et al. simply conjugated the PSCA mAb with QD605 to develop a useful targeted fluorescent probe [73]. This probe could recognize bladder cancer cells specifically and emit stable and long-duration fluorescence. Carbonic anhydrase is a kind of tumor biomarker associated with tumor progression and metastasis [74]. Researchers developed CdSe/ZnS QD nanoprobes modified with carbonic anhydrase inhibitors. Notably, the nanoprobes were coated with DHLA-EDADA ligand and glucose residues, ensuring the stability of CdSe/ZnS QD, as well as providing good biocompatibility and dispersion [75].

Heteroatom-doped graphene quantum dots (GQDs) might be a promising luminescent material that could emit white light and broad excitation-dependent full-color photoluminescence from 463 nm to 672 nm. In addition, it exhibited a prohibiting growth and adhering effect when treating bladder cell lines [76]. However, the growth of tumor cells in vivo is different from that in culture dishes, and the result of inhibiting bladder cancer cell proliferation may be difficult to repeat in animal models.

**Figure 4:**
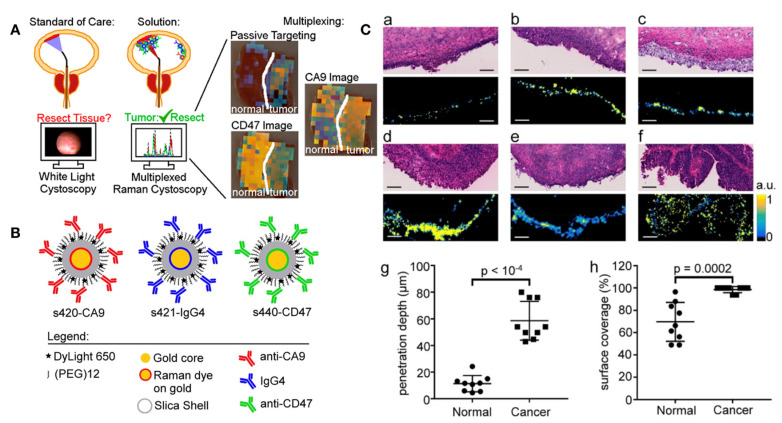
Surface-enhanced Raman nanoparticle could simultaneously carry a variety of antibodies which enabled the multiplexed detection of multiple molecular targets. (**A**) Surface-enhanced Raman nanoparticle endoscope system with multiplexed molecular imaging of CD47 and CA9 enabled to classification of bladder tissue as normal or cancerous. Multiplexed molecular imaging of CD47 and Carbonic Anhydrase 9 tumor proteins. (**B**) The antibodies bound different color surface-enhanced Raman scattering (SERS) nanoparticles. The blue IgG4 nanoparticle is used as a negative experimental control for the active binding of CA9- and CD47-targeted SERS nanoparticles. (**C**) H&E stains (**top**) and s421-IgG4 Raman images (**bottom**) of normal bladder tissue (**a**–**c**) and different grade of bladder cancer tissue (**d**–**f**). (**g**–**h**): Passively targeted nanoparticles penetrated 5-fold deeper and bound to tumor tissue at 3.3-fold higher concentrations in cancer compared to normal bladder urothelium. Reprinted with permission from ref. [77]. Copyright 2018, ACS.

Though the existing fluorescent cystoscope can target bladder cancer cells more precisely, given the molecular heterogeneity of tumors, it is unable to detect 100% of tumor cells with a single target. Therefore, Davis et al. developed a new endoscope system based on surface-enhanced Raman scattering nanoparticles [77]. This Raman nanoparticle could simultaneously carry a variety of antibodies which enabled the multiplexed detection of multiple molecular targets. With the help of multiple antibody-based targeting as well as EPR effect of nanoparticles, Raman nanoparticles could accumulate in bladder tumor tissue and enable multiple imaging (Figure 4).

Imaging cancer cells by specifically targeting overexpressed biomarkers is a good prospect in tumor diagnosis. However, the spectrum used for optical imaging should have the characteristics of low light scattering, minimum tissue absorption, and low spontaneous fluorescence. Near-infrared fluorescence possesses the characteristics of low light scattering, low light absorption and low spontaneous fluorescence when irradiating tissue. Therefore, it has a better tissue penetration ability and enables the imaging of deeper tissues. In order to diagnose tumors with near-infrared rays (NIR), NIR probes and imaging systems that can target tumors are necessary. Cho et al. developed nanoclusters of up-conversion nanoparticle (UCNP) conjunct with antibodies to epidermal growth factor (EGFR). This UCNP could emit visible light under NIR irritation and produce high-contrast images with no background fluorescence, hence accurately indicating the location of cancer cells [78].

### 4.2. Nanotechnology in Urine Test

Urine test for diagnosis of bladder cancer includes urine cytology and biomarkers detection. Urine exfoliative cytology detection mainly identifies abnormal cells in urine. Although it has a high sensitivity to advanced cancer, it possesses poor sensitivity to low-grade cancer. Therefore, it is much better to use high-sensitivity and -specificity nanotechnology to diagnose bladder cancer by detecting urine biomarkers.

A new background-eliminated fluorescence platform for sensitive and selective detection of cancer biomarkers has been built by combining feroxyhyte nanosheets (δ-FeOOH) with amino-functionalized silicon quantum dots (Si QDs). Using highly expressed hyaluronidase (Haase) as the target to detect the urine of bladder cancer patients, this platform possessed a similar accuracy compared to ELISA. The results showed the detection limit for Haase was 0.02 ng/mL (based on 3σ/S), which was three orders lower than most of the reported fluorescence biosensors (Table 2) [76]. Telomerase is an important biomarker in bladder cancer diagnosis. Ma et al. developed a highly sensitive ratiometric fluorescence paper sensor to detect telomerase in urine based on the color change of Rox-DNA functionalized QD. With the help of telomerase, H_2_O_2_ turned into H_2_O and O_2_, and the detected color, therefore, changed from red to yellow or green, which enabled visual semi-quantitative detection with naked eye. More importantly, the detection limit was 10 cells and response time was within an hour [79].

## 5. Nanotechnology in Treatment

### 5.1. Nano-Formulations for Chemotherapy

Paclitaxel (PTX) and docetaxel (DTX) are commonly used chemotherapy drugs. It is often used in systemic chemotherapy and neoadjuvant chemotherapy in the treatment of bladder cancer. However, the poor solubility in water and large systemic adverse reactions after intravenous dripping bring pain to the patients. Therefore, in order to improve the solubility and dispersion of PTX, accurate drug delivery and reduced adverse reactions via nanomaterials is very promising.

Nanoparticle albumin-bound (nab)-paclitaxel is a solvent-free formulation of paclitaxel approved for the treatment of metastatic breast cancer. Compared with standard PTX, nab-paclitaxel is better tolerated and demonstrated better efficacy than standard taxanes [80,81]. Nab-paclitaxel has also shown great efficacy on bladder cancer in the United States and Canada’s clinical trials [82,83] (Table 3).

Lumbrokinase (LK) is an enzyme complex with anti-thrombotic and antitumor effects, which may be promising to cure tumors with chemo drugs. Nevertheless, the toxicity and side effects of chemo drugs and LK limit clinical application. Hu et al. skillfully used block copolymer PEG-b-(PELG-g-(PZLL-r-PLL)) as a nanocarrier to deliver lumbrokinase (LK) with PTX and avoided the great toxicity and ensure sustained release in bladder. In addition, LK/PTX/PEG- b-(PELG-g-(PZLL-r-PLL)) successfully suppressed bladder tumor growth and induced bladder cancer cell death both in vivo and in vitro [84].

Pan et al. developed disulfide-crosslinked PLZ4 nanomicelles (DC-PNM) to deliver PTX. The loading efficiency of PTX was more than 99% and PTX was able to be released under the physiological concentration of glutathione. Interestingly, after being decrorated with PLZ4, the nanoparticles could target bladder tumors specifically but did not accumulate in the lung cancer xenografts, which could greatly reduce the systemic toxicity caused by PTX chemotherapy [85]. This nanomedicine has entered a phase 1 clinical trial in the United States (NCT05519241). 

Liu et al. used chitosan (CS) nanosuspensions (NSs)-capsuled PTX, which exhibited a sustained and prolonged delivery as well as improved the curative effect of PTX intravesical bladder cancer. The positively charged properties enable the PTX/CS NSs to easily attach to the inner mucosa of the bladder through electrostatic adsorption and the nanoparticles could prolong the release of PTX for more than 10 days [86].

Ephrin receptor A2 (EphA2) is a kind of receptor tyrosine kinase that can regulate intercellular interactions and differentiation [87]. It has been found that EphA2 is highly expressed in bladder cancer patients and was associated with progression. EphA2 is a membrane-bound protein and is easy to internalize after antibody binding, which is a hopeful target for drug development. Walid Kamoun et al. developed a new EphA2 targeted antibody-directed nanotherapeutic drug (ADN), named EphA2-ILS-DTXp, which encapsulates the hydrolysis-sensitive prodrug of docetaxel (DTXp) [88]. EphA2-ILS-DTXp was designed to possess good pharmacokinetic properties to reduce plasma exposure while still maintaining the selective tumor exposure of DTX. The results showed that EphA2-ILS-DTXp was far more effective than a combination of free docetaxel and gemcitabine in the treatment of EphA2 positive patient-derived xenograft (PDX) model of bladder cancer [89].

MMC has good water solubility. Although it is convenient for intravesical instillation, it is difficult for it to penetrate the lipophilic cell membrane, therefore the curative effect is often unsatisfying. It has been reported to deliver MMC through a Mn:ZnS QD and chitosan-based nano-delivery system with excellent internalization efficiency which could improve the bioavailability of MMC [90].

**Figure 5:**
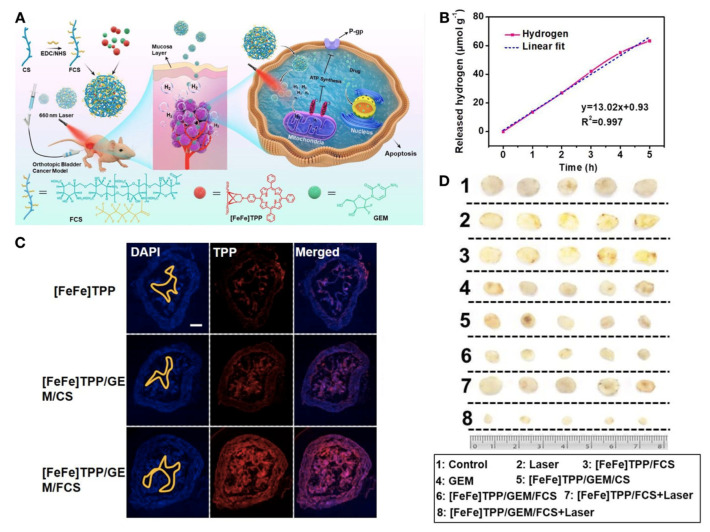
(**A**) A photoactivated H_2_ nanogenerator that comprises an FCS, gemcitabine, and a catalyst of H_2_ production ([FeFe]TPP). Under laser irradiation of 660 nm, the hydrogen catalyst could effectively generate non-toxic hydrogen which could enhance the effect of gemcitabine. (**B**) The gel could release hydrogen gradually. (**C**) After instillation with each formulation, the bladder frozen section experiment as performed (([FeFe]TPP): red fluorescence). ([FeFe]TPP)/GEM/FCS possessed a good tissue penetration ability. (**D**) The T24 cells were planted into the bladders of nude mice. After 4 weeks of treatment, the tumors were harvested. The tumors dramatically shrunk after treatment with ([FeFe]TPP)/GEM/FCS and the laser. Reprinted with permission from ref. [91]. Copyright 2020, ACS. FCS: Fluorinated chitosan; GEM: gemcitabine; TPP: tetraphenylporphyrin; FeFe: a hydrogenase subsite analogue [Fe_2_S_2_(CO)_6_].

Gemcitabine is one of the most commonly used chemo-drugs for bladder cancer. Fluorinated chitosan (FCS), gemcitabine, and H_2_ production catalyst were creatively assembled into a nano-drug delivery platform. In this research, fluorinated chitosan was used to enhance the ability to cross bladder mucosa and penetrate tumor cells. At the same time, under 660 nm laser irradiation, the hydrogen catalyst could effectively generate non-toxic hydrogen. The results showed that hydrogen could inhibit the mitochondrial function of bladder cancer cells, thus inhibiting the P-glycoprotein from excreting gemcitabine (Figure 5) [91].

Qiu et al. combined PAMAM, a kind of dendrimer, with PEG to form the PEG–PAMAM complex as a nanocarrier to deliver Doxorubicin (Dox). The results showed that PEG–PAMAM was a stable nanocarrier with a small size and good biosafety. Dox is released slowly from PEG–PAMAM at a neutral environment while released rapidly in an acidic microenvironment. As a drug carrier, PEG–PAMAM could effectively penetrate the urothelium of the mouse bladder, and increase the content of DOX in the bladder wall after intravesical instillation [92]. Their work provided a new idea for bladder cancer drug delivery.

Cisplatin is often used in systematic chemotherapy. Ding et al. [93] developed multifunctional BITT@BSA-DSP nanoparticles which could deliver cisplatin while producing NIR fluorescence imaging. In addition, with infrared irradiation, this multifunctional nanoparticle can be administrated precisely into the tumor. At the same time, it showed good photothermal and photodynamic effects, which enhanced the sensitivity of cancer cells to cisplatin and reduced the drug resistance in vivo.

In addition to the conventional chemotherapeutic drugs mentioned above, some natural components have also been reported to be used for chemotherapy. β−Elemene (β−E) is an anti-tumor chemical extracted from the herb *Curcuma wenyujin* [94]. So far, elemene emulsion injection as well as oral emulsion have been reported to be used to treat various cancers [95]. Zhai et al. developed an amino-terminal fragment (ATF) peptide-targeted liposome to carry β-elemene (ATF24-PEG-Lipo-β-E). ATF24-PEG-Lipo-β-E had stable shape and size. Additionally, urokinase-type plasminogen activator receptor (uPAR) tends to overexpress in high metastatic cancer cells and tumor stromal cells [96]. ATF peptide can compete to bind uPAR on the surface of tumor and endothelial cells, so as to inhibit tumor growth and angiogenesis, which therefore could help liposomes target delivery to tumors and stromal cells. The results suggested ATF 24−PEG−Lipo−β−E combined with cisplatin significantly inhibited the proliferation of bladder cancer cells in vivo [97].

Polybia-mastoparan I (MPI) is a kind of antibiotic peptide isolated from the venom of the social wasp *Polybia paulista* [98]. In previous studies, MPI showed potent antiproliferative effects and might act as a potential agent for bladder cancer treatment [99]. Li et al. synthesized fluorinated PEI, a transmucosal agent, and conjugated it with MPI to form a bladder instillation formula. Not only did this nano-formula possess great selectivity and biosafety, but also showed a great curative effect on bladder tumors [100].

Azurite ore is a kind of natural copper mineral with a potential anti-tumor effect. Xiong et al. synthetized Cuprous oxide nanoparticles (CONPs) with azurite ore and these water-soluble NPs showed remarkable anti-tumor effects in several cancers. After internalizing by bladder cancer cells, CONPs induced ROS generation, thus causing autophagy and apoptosis [101].

The enzyme/prodrug system is another method for cancer therapy. Indole-3-acetic acid (IAA) is well-known as a plant hormone. Significantly, IAA can be oxidized by horseradish peroxidase (HRP) and then induces the generation of ROS as well as lesions of DNA [102]. A CS/HA NP capsuled with IAA or HRP was developed and reduced the cell viability of the human bladder cancer cell line (T24) by 88% [103]. The hyaluronic acid (HA) mounted on the surface enables a specific adherence to bladder cancer cells. However, in order to avoid being oxidized prematurely, IAA and HRP were capsuled separately, and nano-formulation has not been tested in vivo yet (Table 4).

### 5.2. Nano-Formulations for Immune Therapy

Despite the positive outcomes of BCG in treating NMIBC, there are concerns regarding the side effects, including bladder inflammation, sepsis, and allergy. In addition, BCG-relapse and BCG-refractory have severely limited their application. Several studies successfully reduced the side effect of BCG therapy and prolonged BCG exposure time in the bladder via nanotechnologies. For example, Zhang et al. developed a magnetic thermosensitive hydrogel formulated with chitosan (CS), b-glycerophosphate (GP), and Fe_3_O_4_ magnetic nanoparticles (Fe_3_O_4_-MNP). This hydrogel could gelate quickly under room temperature and deliver BCG to the bladder mucosa. Under an applied magnetic field, it could prolong intravesical BCG residence time [104].

BCG cell wall skeleton (BCG-CWS) is the key component of BCG and a promising substitute for BCG, which has the same immunogenicity as BCG and would not cause tuberculosis infection [105]. However, the insolubility has limited its use. Harashima capsuled BCG-CWS nanoparticles (CWS-NP) by liposome evaporated emulsified lipid (LEEL). Both in vitro and vivo, the CWS-NP could be internalized by bladder cancer cells and inhibit cancerous cell growth [106,107]. Due to the excellent water dispersibility, it has been intravenously injected into advanced cancer patients as an immune adjuvant [108]. On this basis, Yoon et al. combined the CWP-NP with folic acid and Pep-1 peptide as targeting and cell-penetrating moieties. With help of these specific ligands, CWS-NP was absorbed intracellularly by bladder cancer cells to a significant level both in vitro and vivo. In addition, dual-ligand-functionalized liposome was also superior to single-ligand-functionalized liposomes when inhibiting cancer growth [109]. Later, Whang et al. revealed that CWS-NP induced bladder cancer cells by activating oxygen species (ROS) generation and AMPK activation [110]. Other studies found that the combination of nanoparticle BCG-CWS and octaarginine-modified liposomes could suppress bladder carcinogenesis. Moreover, compared with the normal BCG instillation, the smaller dose obtained better efficacy [111,112,113] (Table 5).

### 5.3. Nano-Formulations for Targeted Therapy

Small interfering RNA (siRNA) is widely used to inhibit the expression of oncogene because of the capacity of degrading mRNA. Several studies have demonstrated that the knockdown of oncogene can inhibit bladder cancer cell proliferation. However, the single-strand structure makes it easily degradable and the negative charge makes it difficult to penetrate cell membrane. Thus, an extra carrier is needed to protect siRNA from degradation.

Lipid or virus vehicles are the most commonly used siRNA delivery system. However, these vehicles are usually cytotoxic in humans. Chen et al. developed a Mg (II)-Catechin nano-gene delivery system to deliver siRNA EIF5A2. Both in the T24 cells and xenogenic model, Mg(II)-Cat/siEIF5A2 complex suppressed bladder cancer growth. In addition, this self-assembled, well-compatible Mg(II)-Catechin NPs showed a preferential uptake by bladder cancer and a good synergistic anti-tumor effect [114]. This study offered a new idea for NPs’ design by using natural anti-tumor compounds to enhance the NPs.

Survivin is an oncogene that can inhibit apoptosis and promote cell proliferation and the over-expression of survivin helps cancer cells to escape from cell cycle checkpoints and inhibits apoptosis [115,116,117]. Thus, it plays an important role in tumor growth and drug resistance [118,119]. Maria et al. used a poly (beta-aminoester) proprietary polymer to encapsulate siRNA survival and tested it in two bladder cancer cell lines. This nanoparticle has a C32 pBAE backbone, including 50% arginines and 50% lysines as terminal oligopeptides, with a coating of the protein bromelain, which makes it easier to cross the mucosal barriers. The bladder cancer cells showed a significant decrease in cell viability after treatment with siRNA NPs, however, the effectiveness of PTX is much better than that of siRNA NPs. Furthermore, a synergistic effect was not observed when using the two anti-tumor agents simultaneously, which indicated these siRNA NPs could not improve PTX resistance of bladder cancer cells [120].

It is difficult for drugs to penetrate bladder tumors due to the bladder permeability barrier (BPB), which consists of umbrella cells, tight junctions, and plaques. In order to solve this problem, Martin et al. used a poly (lactic-co-glycolic acid; PLGA) NP to transport siRNA survivin. Low-molecular-weight positively charged mucoadhesive chitosan was added to the surface. Chitosan can adhere to bladder urothelium hence prolonging the NPs’ retention time in the bladder [121]. With the enhancement of AP as well as low-molecular-weight chitosan, the intake rate of these NPs in mouse bladder and human ureter tissue was 10 times than the level of unmodified PLGA NPs. SiRNA release time was prolonged and the volume of the bladder tumor in the xenograft model shrunk significantly [122].

AIB1 was initially found to be over-expressed in many cancers. Subsequently, it was shown to be expressed in many human urothelial carcinomas of the bladder and is a new independent prognostic marker of patient survival [123,124]. Wei et al. used ACC/CaIP6 nanocomposite particles (NPACC/CaI--P6 to transfer siRNA AIB1 [125]. This novel nanoparticle was not cytotoxic. Both in vitro and in vivo, this ACC/CaIP6/siRNA complex cloud inhibits cell proliferation, induces apoptosis induction and down-regulates the PI3K/Akt pathway (Table 6).

### 5.4. Nano-Formulations for Light-Based Therapy

The novel nanotechnology may help the broader application of PDT therapy for the treatment of bladder cancer. Researchers developed various nanomaterials which possess much better selectivity to the tumor and then loaded these with photosensitizers. Hopefully, through these enhancements, dyes will be able to highly concentrate in tumors rather than adjunctive tissue and be retained in the bladder for a longer time. In general, these nanomaterials can be divided into two types. The first type, active targeting, can attach to bladder cancer cells or the surface receptors actively by modifying the nanomaterials with ligands and antibodies, etc. Because of the improvement of drug selectivity, the curative effect can be enhanced while the adverse events can be reduced in theory. The other type, passive targeting, mainly passively penetrates bladder cancer tissue by taking advantage of the enhanced permeability and retention (EPR) effect.

The synergistic effect of porphyrins and graphene-based nanomaterials in PDT has been reported, in which graphene oxide (GO) and graphene QDs are used as drug carriers [126,127]. These nanomaterials not only have a larger surface area to carry the photosensitizer, but also can improve the stability of the photosensitizer in liquid. Luca et al. non-covalently loaded three different photosensitizers (PS) (TMPyP, Zn-TMPyP, and P1-C_5_) on GO or GQD in one step. This porphyrin delivery system could respond to blue and red light simultaneously and showed great cytotoxic activity against T24 cell lines [128]. Moreover, this material can absorb NIR and generate heat, which can prompt further study into the possibility of their use in PTT.

The hypoxic environment caused by tumors and PDT can significantly decrease the effect of PDT. Wu et al. mixed the conventional antiparasitic agent nitazoxanide (NTZ) with chlorine e6 (Ce6)-conjugated human serum albumin (HSA), and HSA–Ce6 is able to form self-assembled HSA–Ce6/NTZ nanoparticles (NPs). Then, the HSA–Ce6/NTZ complexes further fabricate with FCS, the synthesized transmucosal carrier, to form a biocompatible nanoscale system HSA–Ce6/NTZ/FCS NPs. These novel nanoparticles could remarkably improve the hypoxic environment of tumors and therefore ensure the sustainability of PDT therapy [129].

The plaques of the glycosaminoglycan mucin layer, as well as the tight cell junctions on the mucosa layer of the bladder bring difficulties to drug penetration. The self-assembled CAT–Ce6/F-PEI nanoparticles, which were obtained by simply mixing fluorinated polyethyleneimine (F-PEI) with Chorin-e6-conjugated catalase (CAT–Ce6), had great membrane penetration ability. Furthermore, it could take advantage of the decomposition of tumor endogenous H_2_O_2_ by CAT–Ce6/F-PEI to improve the hypoxic environment, and enhance the PDT effect. The in vivo experiment proved the great antitumor activity, as well as the low systemic toxicity compared to the common photosensitizer [130]. Luo et al. constructed a nano-drug delivery platform poly (OEGMA)-PTX@Ce6 (NPs@Ce6) by combining photosensitizer Ce6 with a cathepsin B-sensitive polymer-paclitaxel (PTX) prodrug [131]. This system used a strategy called photochemical internalization (PCI), which basically contained the nano-capsuled photosensitizers. This system can take advantage of the ROS generated by photosensitizers to increase the permeability of cell membranes and then increase the uptake of nanoparticles [132,133]. The results both in T24 cell lines and the xenograft model suggested that photodynamic treatment at a low dose could promote cellular uptake of NPs@Ce6 and enhance their spheroid penetration [131].

It has been reported that persistent PDT can cause the regulation of PD-L1, which can help cancer cells escape, hence decreasing the effect of PDT. To mitigate the impact of PD-L1 upregulation, current studies have combined PDT with immune checkpoint drugs via nanotechnology. Metformin (Met) is much cheaper than PD-1/PD-L1 antibodies but can unexpectedly reduce the expression of PD-L1 [69,134,135]. In order to transport Met into bladder cancer cells and prolong drug retention time, researchers loaded Met and IR775 into a clinically usable liposome as a two-in-one nanoplatform (IR775@Met@Lip), successfully decreasing the expression of PD-L1 in a bladder cancer cell and reversing tumor hypoxia suppression simultaneously, therefore improving the curative effect of PDT [69]. Chin et al. also developed a special nanoparticle to conduct PDT therapy as well as immune activation, which includes lowering the PD-L1 signal [136]. Using Fe_3_O_4_ and iron chlorophyII (Chl/Fe) as photosensitizers showed a satisfying photodynamic effect. In addition, 4-carboxyphenylboronic acid (CPBA) on the surface enabled NPs to target the BC wall through glycoproteins and the iron-induced ferroptosis of bladder cancer cells. The reduction in PD-L1, TGF-β, IDO-1andM2-like macrophages demonstrated the possibility of reprograming the TME from cold (immune depletion) to hot (immune activation).

Under the irradiation of a specific wavelength laser, photothermal agents can convert light into heat, raise the temperature, and thus kill tumor cells. Photothermal therapy (PTT) is simple, non-invasive, and safe, which makes it a promising therapy in tumor treatment. Various inorganic nanomaterials and polymers have been used for PTT. Intravesical instillation is often used in PTT. Although it is convenient, the short retention time of the drug and the poor permeability greatly affects the curative effect and application of PTT. Compared with visible light and near-infrared-I, near-infrared-II (NIR-II) significantly increases the tissue penetration depth of light-based therapy. Based on this, Zhang et al. developed FCS-capped Cu_2-x_Se nanoparticles which were prepared through the one pot aqueous method. In addition, due to the excellent mucosal penetration capacity of FCS, the FCS-enhanced photothermal conversion agent Cu_2-x_Se shows a better penetration effect, which remarkably improved the therapeutic effect of NIR-II PTT for orthotopic bladder cancer [137]. TiO_2_ is another commonly used metallic oxide material for PTT. It is reported that the black TiO_2_ with a narrow band gap of 2.32 eV from P25 reduction exhibited a great PTT effect under 808 nm light triggering [138].

Some nanoparticles can produce a large amount of ROS as well as heat under NIR, that is, taking advantage of PDT and PTT simultaneously. Yu et al. developed spherical pheophorbide a-hydrazone-doxorubicin nanoparticles (PhD) which carried DOX inside the nanospheres. After being injected into a vein, these NPs accumulate in tumor tissue through EPR effect. Disassembly ensued when NIR was irradiated or in the low pH environment. Meanwhile, Pheophorbide possessed photodynamic and photothermal effects under NIR. Together, not only did this triple-model therapy realize an anti-tumor activity in vivo and in vitro, but also demonstrated a well potential to overcome chemo-drug resistance [139].

**Figure 6:**
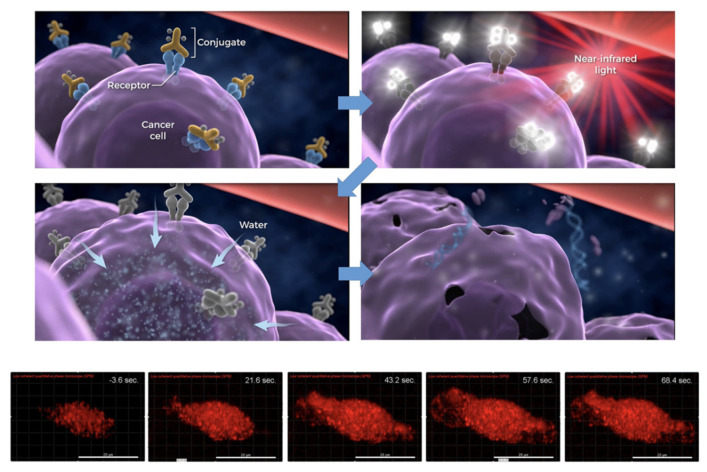
The diagram of near-infrared photoimmunotherapy (NIR-PIT). The targeted photosensitizer can better distinguish tumor cells therefore PIT can destroy tumor cells more selectively under the NIR laser. Reprinted permission from ref. [140]. Copyright 2019, ACS.

Near-infrared photoimmunotherapy (NIR-PIT) is a kind of brand-new photodynamic therapy, which can recognize tumor cells by targeted photosensitizers. Only when the photosensitizer combines with tumor cells can it be activated by light and initiate the phototoxicity. Compared with the traditional photosensitizers, targeted photosensitizers can better distinguish tumor cells, hence PIT can destroy tumor cells more selectively without damaging normal tissues [140] (Figure 6).

A novel multi-responsive mesoporous polydopamine composite nanorods were designed to carry indocyanine green (ICG) and PD-L1 antibodies. The wheat germ agglutinin was added to the surface of this nanorod which enabled the specific adherence to epithelial glycocalyx of the bladder cancer cell, thus decreasing the injury of normal tissue. In addition, MnO_2_, the skeleton of this nanoparticle, could induce disintegration of H_2_O_2_ and therefore improve the hypoxia. The 2,3-dimethylmaleic anhydride on the nanoparticles brought out the slow release of PD-L1 antibodies under the low pH, therefore it possessed the PTT and anti-PD-L1 efficacy without damaging the normal tissue [141].

**Figure 7:**
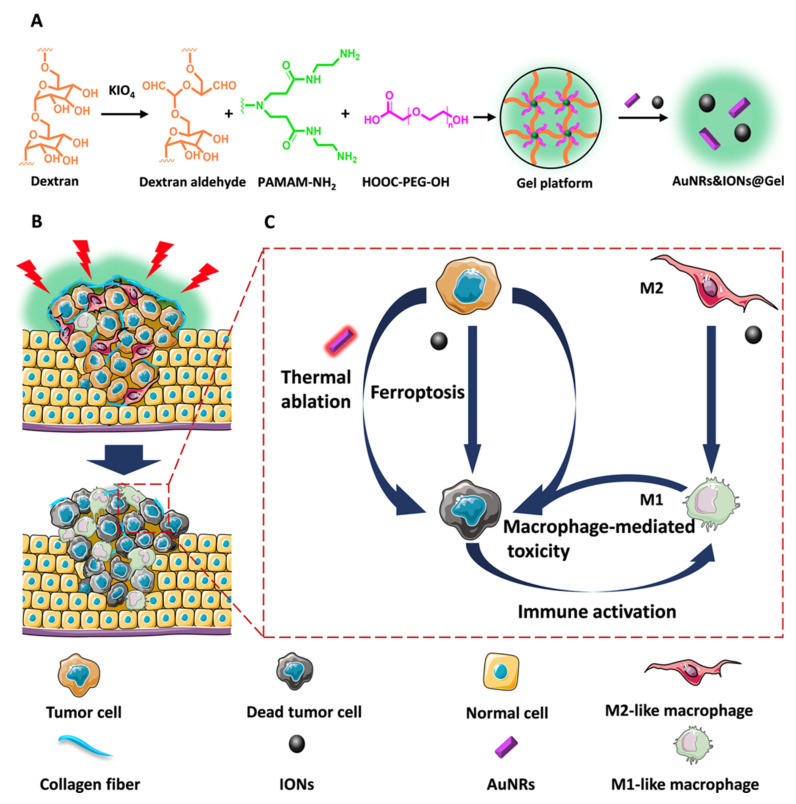
Multiple-function AuNRs&IONs@Gel can target bladder tumors and perform accurate PTT with the help of nano-particle imaging. In addition, the ion can induce iron death as well as modulate TME, enhancing the curative effect. (A) Schematic illustration of how the AuNRs&IONs@Gel was synthesized. (B, C) AuNRs&IONs@Gel-induced ICD through triple therapy. Reprinted with permission from ref. [142]. Copyright 2020, ACS.

Guo et al. developed AuNRs&IONs@Gel, a multiple-effect nanoparticle, which consists of a gel delivery platform, embedded gold nanorods (AuNRs), and iron oxide nanoparticles (IONs), in order to relieve tumor drug resistance. Firstly, the gel delivery platform can target the glucan aldehyde of collagen which is overexpressed in tumor tissue. In this case, the AuNRs perform imaging and accurate photothermal transformation under near-infrared radiation to ablate tumor tissue. Then, a high concentration of ions can be absorbed and induce the iron death of tumor cells. Last but not least, tumor-associated macrophages, which are usually M2-like phenotypes, will be repolarized by ions into M1-like phenotypes, playing the roles of anti-tumor and antigen presentation agent [142] (Figure 7).

CD47 is also known as integrin-associated protein (IAP), which is highly expressed on the surface of bladder cancer cells and protects them from being phagocytized by interacting with the SIRP protein mounted on the NK cell surface. Under the NIR, Bernhard et al. significantly slowed bladder cancer cell growth with anti-CD47-IR700. Notably, the retention time of anti-CD47-IR700 in bladder tumor tissue was up to at least two weeks, which might reduce the frequency of bladder instillation and reduce the pain of it [143].

CD 44 (HA receptor) is highly expressed on the surface of the bladder cancer cells. Self-assembled, HA-enhanced nanomaterials were synthesized in order to carry IR780. Significantly, the NPs could be degraded by HAase when it was over-accumulated in normal bladder cells, therefore exhibiting excellent biocompatibility [144] (Table 7).

### 5.5. Nano-Formulations for Sonodynamic Therapy

With the application of PDT and PTT in tumor treatment, their shortcomings are gradually revealed, such as the poor penetration of visible light, and the phototoxicity caused by long-term retention of photosensitizer in skin tissue, etc. These shortcomings limit the use of light-based therapy in the tumor, while sound-based therapy overcomes the main limitations of light-based therapy to a great extent. Yumita et al. discovered the cytotoxicity of hematoporphyrin in the sound field for the first time and defined it as sonodynamic therapy (SDT) [145]. Sonodynamic therapy has the advantages of deeper tissue penetration, better precision, fewer adverse reactions, and good patient compliance. Nano-acoustic sensitizers can significantly improve the effect of acoustic dynamics in tumor treatment. Li et al. developed Catalase-Meso-tetra(4-carboxyphenyl) porphine-fluorinated chitosan nanoparticles (CAT-TCPP/FCS NPs), a self-assembled SDT formula that possesses remarkable tumor tissue penetration abilities and SDT effects. In addition to the penetration of FCS and the cytotoxicity of sonosensitizers, the catalase-catalyzed O_2_ generation from tumor endogenous H_2_O_2_ relieves hypoxia, enhancing the curative efficacy [146].

**Figure 8:**
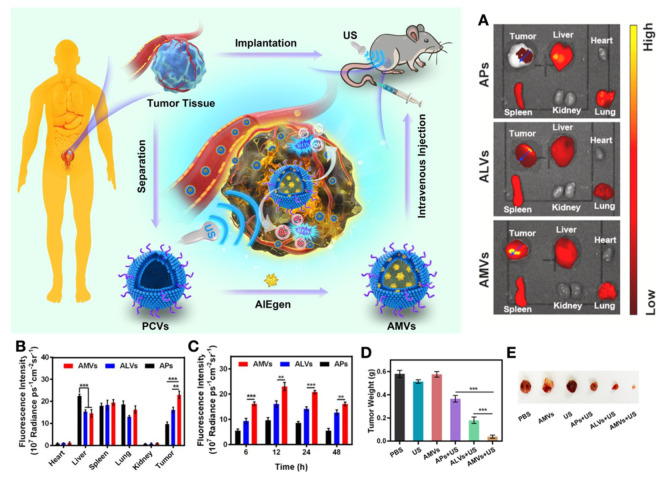
A patient-derived MVs/AIEgen hybrid system (AMVs) for personalized SDT in bladder cancer patient-derived xenograft (PDX) models. (**A**) Ex vivo images of excised organs and tumors of mice bearing PDX were examined at 12 h post-injection of APs, ALVs, and AMVs. (**B**) Fluorescence distribution profile on the specified blue arrows in tumors of (**A**). (**C**) Quantification of the time-dependent fluorescence intensity at the tumor site after various treatments. (**D**) Tumor weight and (**E**) tumor tissues were collected from the PDX mice after various treatments. APs, ALVs, and AMVs: PLGA/AIEgen nanoparticles; ALVs: MVs/AIEgen derived from a cell line. ** *p* < 0.01; *** *p* < 0.001. AMVs: patient-derived MVs/AIEgen hybrid system. Reprinted with the permission from ref. [147]. Copyright 2021, Elsevier. MVs: microvesicles; PDX: patient-derived xenograft; SDT: sonodynamic therapy; APs: PLGA/DCPy hybrid nanoparticle; ALVs: T24 cell-derived MVs/AIEgen hybrid system; AMVs: patient-derived MVs/AIEgen hybrid system; US: ultrasound.

Recently, aggregation-induced emission (AIE) has been widely studied because of its better photostability and biocompatibility, and AIE-based sonosensitizers have been applied for SDT. Duo et al. combined microvesicles with AIE molecules to develop AIE-active sonosensitizers (AMVs) and demonstrated superior targeting and personalized SDT for bladder cancer [147]. Remarkably, their results demonstrated a personalized tumor targeting ability, which possessed possible applications for subsequent personalized SDT (Figure 8) (Table 8).

## 6. Conclusions and Perspectives

Nanomaterials can easily carry various tumor biomarkers or fluorescent materials, making biomarker detection no longer limited to ELIIS and immunohistochemistry, etc. In addition, compared with traditional cystoscopy, CT scan, and urine test, the nanomaterials for bladder cancer diagnosis have the advantages of excellent selectivity as well as in situ visualization. Therefore, the combination of common diagnostic methods and nanomaterials can greatly improve diagnostic efficiency. At the same time, the advanced nano-diagnostic probes enable doctors to conduct accurate visual treatment. In addition, the combination of therapeutic agents on a single nanoplatform can realize diagnosis, treatment and curative effect monitoring simultaneously, which emphasizes nanoparticles’ huge potential in bladder cancer diagnosis. However, current research studies on nanotechnology for bladder cancer diagnosis often lack data on sensitivity and specificity, which are critical indicators to evaluate the effectiveness of diagnostic methods. In addition, it has not been reported that the individual tumor characteristics, tumor stage and other parameters of nanomaterials can be identified. Thus, further research is needed to design nanoparticles that can diagnose the nature, size, and depth of tumors more accurately. Furthermore, compared with therapeutic nanoparticles, diagnostic nanomaterials are easier to test in clinical practice. Nevertheless, none of them have entered the United States clinical trials as of yet, hence the clinical application of diagnostic nanoparticles should be promoted.

Nano-treatment of bladder cancer depends on chemo-drugs, immune drugs, and targeted drugs, as well as photosensitizers and acoustic sensitizers. With the in-depth study of drug delivery, nano-drug delivery platforms stand out among various drug carriers. Their great advantages in drug release, stability, and biocompatibility have been widely recognized by researchers. Nanomaterials attach to the tumor mainly through EPR effect and active targeting. However, the heterogeneity of the vascular system, the different tumor types, as well as the variability of the tumor microenvironment, make EPR uncertain. For this reason, researchers have used new targeting strategies when designing nanoparticles, such as equipping the surface of nanoparticles with proteins that can specifically bind to tumor cells, using metal and magnetic particles to realize visible and controllable transport, etc.

In addition to increasing delivery efficiency and improving drug distribution, an excellent nano-drug delivery platform requires a controllable drug release process, which will also be of benefit to reducing the side effects of treatment. It is an effective strategy to design tumor environment response nanoparticles, so as to take advantage of the acidic, hypoxic environment, etc. Last but not least, the nano-platform can carry multiple therapeutic agents at the same time, and the integration of multiple therapeutic methods will undoubtedly ease the drug resistance of tumors. Therefore, the development of multiple anti-tumor effect nanoplatforms will be the trend in the future. The research on nanomedicine in treating bladder cancer has bloomed in the past decades. Some nanomedicines such as Nab-paclitaxel demonstrated promising results in other countries’ clinical trials, but only two nano-formulations entered the clinical trials in United States and none of them were approved by FDA.

In general, these interesting outcomes of experimental research as well as clinical tests reveal the broad prospects of nanotechnology. The command of efficient diagnostic nanomaterials and safe therapeutic nano-drugs will be consistently drawing attention for the years to come.

## Figures and Tables

**Table 1:** FDA-approved biomarkers.

Biomarkers and Manufacturer	Detected Biomarkers	Assay Type	Specimen	Sensitivity (CI 95%)	Specificity (CI 95%)	Ref.
NMP22 (Matritech, Inc., Alere, Jena, Thuringia, Germany)	Nuclear mitotic apparatus proteins	ELISA	Urine	62–75%	70–83%	[31]
NMP22 (Matritech, Inc., Alere, Jena, Thuringia, Germany)	Nuclear mitotic apparatus proteins	Point- of-care test	Urine	52–59%	87–89%	[31]
BTA Stat (Polymedco, Cortlandt, NY, USA)	Complement factor H-related protein and complement factor H	Point-of-care test	Urine	58–69%	73–81%	[31]
BTA TRAK (Polymedco, Cortlandt, NY, USA)	Complement factor H-related protein and complement factor H	ELISA	Urine	54–75%	64–82%	[31]
UroVysion (Abbott Vysis, Chicgo, Illinois, USA)	Alterations in chromosomes 3, 7, 17, and 9p21	FISH	Urine	65–84%	78–92%	[32]
uCyt+/Immunocyt (Scimedx, Inc., Dover, New Jersey, USA)	Bladder tumor cell associated mucins/carcinoembryonic antigen	Immunocytochemistry	Urine	78–90%	77–87%	[31]

ELISA: enzyme-linked immunosorbent assay; FISH: fluorescence in situ hybridization.

**Table 2:** Nano-prognostic methods for bladder cancer.

Nanomatierials	Detect Target	Properties	Sensitivity (CI 95%)	Specificity (CI 95%)	Applcations	Ref.
QD625	CD47	High sensitivity and specificity.	82.9%	90.5%	Targeted fluorescent probe for cystoscope	[70]
QD605	PSCA	Specifically targets BC cells and emits stable and long duration fluorescent	-	-	Targeted fluorescent probe for cystoscope	[73]
CdSe/ZnS QD	Carbonic anhydrase	Well biocompatibility and dispersion.	-	-	Targeted fluorescent probe for cystoscope	[74,75]
Heteroatom-doped graphene QD	Haase	Emits white light and broad excitation-dependent full-color photoluminescence from 463 nm to 672 nm.	-	-	Targeted fluorescent probe for cystoscope	[76]
Surface-enhanced Raman scattering nanoparticle	Carbonic anhydrase9, CD47	Multiple targets and imaging	(ROC AUC: 0.95)	-	Targeted fluorescent probe for Raman endoscopy	[77]
UCNP	EGFR	Well ability of tissue penetration	-	-	NIR probe and imaging system	[78]
Si QDs/HA-δ-FeOOH	Haase	Detection limit for Haase: 0.02 ng/mL (based on 3σ/S). RSD < 3% (Compared with ELISA method)	Detection limit: 0.02 ng/mL	-	Fluorescence platform for urine test	[76]
Rox-DNA functionalized QD	Telomerase	Enabled visual semi-quantitative detection with naked eye. The detection limit was 10 cells and response time was within an hour.	-	-	Sensitive ratiometric fluorescence paper sensor	[79]

CI: confidence interval; ROC: receiver operating characteristic; AUC: area under curve.

**Table 3:** Clinical trials for bladder cancer with nano-formulations in the United States.

Nanoparticle	Therapeutic Agents	Condition	Sponsor/Collaborations	States	Study Start	NCT Number
Paclitaxel albumin-stabilized nanoparticle (Nab-paclitaxel)	PTX	Recurrent BC; Stage IV BC	Mayo Clinic/NCI	Phase 2 (Withdrawn)	June 2016	NCT02718742
Paclitaxel albumin-stabilized nanoparticle (Nab-paclitaxel)	PTX	Bladder cancer	University of Michigan Rogel Cancer Center/Celgene Corporation	Phase 2	December 2007	NCT00585689
PLZ4-coated paclitaxel-loaded micelles (PPM)	PTX	NMIBC	VA Office of Research and Development/University of California, Davis	Phase 1 (Not yet recruiting)	-	NCT05519241

BC: bladder cancer; NCI: National Cancer Institute.

**Table 4:** Nano-formulations for chemotherapy.

Nanoparticle	Size (nm)	Therapeutic Agents	Loading Efficiency	Properties	Application	Ref.
Nab-paclitaxel	150–200	PTX	10%	Low side-effects; good solubility and biocompatibility	Vein injection	[80]
LK/PTX/PEGb- (PELG-g-(PZLL-r-PLL))	89 ± 3	LK, PTX	LK (6.74%), PTX (4.13%)	Increasing of the half-life and bioavailability of the drugs	Abdominal subcutaneous injection	[84]
DC-PNM-PTX	23 ± 6	PTX	>99%	Specifically targeting the bladder cancer PDXs; improvement of the cisplatin resistance; GSH-responsive release	Tail vein injection	[85]
PTX/CS NSs	194.48 ± 86.24	PTX	81.4%	Attaching to mucosa of the bladder through electrostatic adsorption	Intravesical instillation	[86]
EphA2-ILs-DTXp	110 ± 10	DTX prodrug	90–99%	Specific targeting to tumor; improvement of penetration; minimal haematological toxicity	Tail vein injection	[88,89]
MMC@CS -Mn:ZnS	175	MMC	44.52 ± 1.05%	Long retention time	-	[90]
[FeFe]TPP/GEM/FCS NPs	220	GEM; [FeFe]TPP	GEM (6.9%); [FeFe]TPP (7.7%)	Improvement of penetration capacity; H2 generation under 660nm laser irradiation; inhibition of drug transport capacity of cancer cells	Intravesical instillation	[91]
PEG-PAMAM-DOX	13	DOX	-	pH-responsive release	Intravesical instillation	[92]
BITT@BSA-DSP	70.2 ± 22.0	DSP	35%	Visible drug delivery; photodynamic and photothermal effect	Intravesical instillation	[93]
ATF24-PEG-Lipo-β-E	79.32 ± 1.282	β-E	98.37%	Specific targeting to tumor	Intravesical instillation	[97]
MPI/F-PEI NPs	260.67 ± 6.62	MPI	-	Improved cross-membrane and transmucosal penetration	Intravesical instillation	[100]
CONPs	40~110	CONPs	-	Activation of ERK-dependent autophagy; synergistic effect with chemo drugs.	Intravesical instillation and in situ injection	[101]
IAA-CS/HA NP and HRP-CS/HA NP	170~200	HRP, IAA	Both > 90%	Enzyme/prodrug system.	In vitro (T24)	[103]

**Table 5:** Nano-formulations for immune therapy.

Nanoparticle	Size (nm)	Therapeutic Agents	Loading Efficiency	Properties	Application	Ref.
Fe_3_O_4_-BCG-CS/GP gel	-	BCG	1% (*w*/*v*)	Response to magnetic field control; long retention time	Intravesical instillation	[104]
CWS-NP/LEEL	166	BCG-CWS	57%	Good water solubility	Intravesical instillation	[106,107]
CWS-FPL	<200	BCG-CWS	60%	Improvement of tumor targeting by folic acid; improvement of penetration by Pep-1 peptide	Intravesical instillation	[109]
R8-liposome-BCG-CW	230	BCG-CWS	-	Improvement of cell binding and internalization	Intravesical instillation	[110]

**Table 6:** Nano-formulations for targeted therapy.

Nanoparticle	Size (nm)	Therapeutic Agents	Loading Efficiency	Properties	Application	Ref.
Mg(II)-Cat/siEIF5A2	10-20	Catechin; siEIF5A2	-	Good biocompatibility and cellular uptake; inhibition of oncogene eukaryotic translation initiation factor	Tail vein injection	[114]
Anti-survivin siRNA-1 pbae-NP	150	Survivin siRNA	100%	No synergistic effect with PTX	In virto (T24, RT4)	[110]
NP-siSUR-CH2.5	137 ± 51	Survivin siRNA	70%	Long release time of sirna	In situ injection	[121,122]
NP-ACC/caip6/siAIB1	80–200	siAIB1	-	Well ability of lysosome escape; good biocompatibility	In situ injection	[125]

**Table 7:** Nano-formulations for light-based therapy.

Nanoparticle	Responsive Part	Size (nm)	Therapeutic Agents	Properties	Application	Ref.
Zn-TMPyP@GQDs	An-TMPyP, GQDs	28.4	-	Blue light-responsive; good stability of porphyrins in aqueous solutions; multiple targets binding sites and possible photothermal effect	In vitro (T24)	[128]
HSA-Ce6/NTZ/FCS	Ce6	192	NTZ	Improvement of tumor hypoxia and drug transmucosal delivery	Intravesical instillation	[129]
CAT-Ce6/F-PEI	Ce6	220.3	-	Improvement of tumor hypoxia by catalase and drug transmucosal delivery	Intravesical instillation	[130]
Poly (OEGMA)-PTX@Ce6 (NPs@Ce6)	Ce6	168.2 ± 1.12	Polymer-PTX prodrug	Combination of PCI effect and enhanced chemo-PDT	In situ injection	[131,132,133]
IR775@Met@Lip	IR775	-	Metformin	Improvement of tumor hypoxia; down-regulate PD-L1	Intravesical instillation	[69]
Fe_3_O_4_@Chl/Fe CNPs	Chl/Fe	12.8 ± 4.8	-	Photodynamic immunotherapy-initiated ferroptosis and immune stimulation.	Intravesical instillation	[136]
FCS-Cu_2-x_Se	Cu_2-x_Se	30.1	-	Improvement of drug transmucosal delivery; NIR-II-responsive	Intravesical instillation	[137]
Black TiO_2_ NPs	TiO_2_	20–30	-	Absorption of visible light and near in- frared	In vitro (T24)	[138]
PhD	Pheophorbide a	71	DOX	Combination of PDT, PTT and DOX; pH and NIR-responsive.	Tail vein injection	[139]
MPDIαW	ICG, MnO_2_	120	PD-L1 antibody	Combination of PTT and immunotherapy; specific adherence to bladder cancer cell; pH-responsive	Intravesical instillation	[141]
AuNRs&IONs@Gel	AuNrs	80–120	Iron oxide nanoparticles	Combination of PTT, iron death, and macrophages re-polarization; targeting delivery	In situ injection	[142]
Anti-CD47-IR700	IR700	-	-	Targeting delivery; long retention time	Tail vein injection	[143]
HA-IR780 NPs	IR780	171.3	-	Targeting delivery; good bioavailability and biocompatibility	Tail vein injection	[144]

**Table 8:** Nano-formulations for sonodynamic therapy.

Nanoparticle	Responsive Part	Size (nm)	Therapeutic Agents	Properties	Application	Ref.
CAT-TCPP/FCS NPs	TCPP	190 ± 12	-	Improvement of tumor hypoxia by catalase and drug transmucosal delivery	Intravesical instillation	[146]
AMVs	AIEgen	300	-	Good internalization and personalized tumor targeting ability	Tail vein injection	[147]

## Data Availability

Not applicable.

## Abbreviations

Abbreviations are listed by the order of first letter.

AIE	Aggregation induced emission
AIE	Aggregation-induced emission
AIEgen	AIE luminogens
ALA	5-aminolevulinic acid
AMPK	Adenosine phosphate activated protein kinase
AP	Penetratin
ATF	Amino-terminal fragment
AUC	Area under curve
AuNRs	Embedded gold nanorods
BCG	Bacillus calmette–guérin
BCG-CWS	BCG cell wall skeleton
BSA	Bovine serum albumin
Cat	Catechin
Cd	Cadmium
CD47	Cytokine
Ce6	Chlorin e6
Chl	Chlorophy
CI	Confidence interval
CS	Chitosan
CT	Computer tomography
CWS-NP	BCG-CWS nanoparticle
Dox	Doxorubicin
DTX	Docetaxel
EGFR	Epidermal growth factor
ELISA	Enzyme linked immunosorbent assay
ELISA	Enzyme linked immunosorbent assay;
EphA2	Ephrin receptor A2
EPR	Enhanced permeability and retention
FAP	Fibronectin attach protein
FCS	Fluorinated chitosan
FDA	Food and Drug Administration
FGFR	Fibroblast growth factor receptors
FISH	Fluorescence in situ hybridization
GC regimen	Gemcitabine cisplatin/carboplatin
GEM	Gemcitabine
GO	Graphene oxide
GP	B-glycerophosphate
HA	Hyaluronic acid
HAase	Hyaluronidase
HLA	Hexaminolaevulinic acid
HSA	Human serum albumin
IAP	Integrin-associated protein
IONs	Iron oxide nanoparticles
LEEL	Liposome evaporated emulsified lipid
LK	Lumbrokinase
mAb	Monoclonal antibody
Met	Metformin
MIBC	Muscle-invasive bladder cancer
MMC	Mitomycin
Mn	Manganese
MNP	Magnetic nanoparticles
MPI	Polybia-mastoparan I
MRI	Magnetic resonance imaging
mRNA	Messenger RNA
MVAC	Methotrexate, vinblastine, doxorubicin and cisplatin
NCCN	National comprehensive cancer network
NIR	Near infrared ray
NIR-II	Near-infrared-II
NK cell	Natural kill cell
NMIBC	Non-muscle-invasive bladder cancer
NSs	Nanosupensions
NTZ	Nitazoxanide
OEGMA	Polyethylene glycol ester
PAMAM	Poly amidoamine
PCI	Photochemical internalization
PCI	Photochemical internalization
PD-1/L1	Programmed cell death 1
PD-L1	Programmed cell death 1 ligand 1
PDT	Photodynamic therapy
PDX	Xenograft
PEG	Polyethylene glycol
PET / CT	Positron emission tomography computed tomography
PLGA	Poly (lactic-co-glycolic acid)
PS	Photosensitizers
PSCA	Prostate stem cell antigen
PTT	Photothermal therapy
PTX	Paclitaxel
QD	Quantum dot
ROC	Receiver operating characteristic
ROS	Reactive oxygen species
SDT	Sonodynamic therapy
Se	Selenium
SI	Singe-dose immediate intravesical chemotherapy
Si	Silicon
siRNA	Small interfering RNA
TCPP	Meso-tetra(4-carboxyphenyl)porphine
TiO_2_	Titanium dioxide
TME	Tumor microenvironment
TURBT	Transurethral resection of the bladder cancer
UCNP	Upconversion nanoparticle
β- E	Β- elemene
δ-FeOOH	Feroxyhyte nanosheets
